# ULK1 in type I interferon response

**DOI:** 10.18632/oncotarget.5241

**Published:** 2015-08-22

**Authors:** Diana Saleiro, Leonidas C. Platanias

**Affiliations:** Robert H. Lurie Comprehensive Cancer Center and Division of Hematology-Oncology, Feinberg School of Medicine, Northwestern University, Chicago, IL, USA

**Keywords:** ULK1, interferon

Type I interferon (IFN) signaling leads to transcription and translation of key IFN-stimulated genes (ISGs), whose protein products exhibit anti-tumorigenic, anti-viral, and immunomodulatory functions [1–3]. These responses are triggered by the interaction of type I IFNs (IFNα, IFNβ, IFNω) with a unique cell surface receptor composed by two subunits: IFNα receptor 1 (IFNAR1) and IFNAR2 [1–3]. After the Type I IFN receptor is targeted by different members of this cytokine family, there is induction of the kinase activities of two known kinases; tyrosine kinase 2 (TYK2) and janus kinase 1 (JAK1), which activate several downstream signaling pathways, including STAT (signal transducer and activator of transcription), MAPKKK (mitogen-activated protein kinase kinase kinase), PI3K (phosphatidylinositol 3-kinase)-AKT, mTORC1 (mammalian target of rapamycin complex 1), and mTORC2 signaling cascades [1–3].

There has been rapid accumulation of information in the IFN-signaling field for over two decades. A detailed map of signaling events and elements that ultimately control IFN-responses has emerged [1–3]. Yet, unique mechanisms accounting for activation and coordination of distinct IFN signaling pathways still need to be defined. Similarly, the elements that account for optimized cellular responses and adjust the balance between negative and positive cellular control in the IFN-response, remain to be established. In a recently published report, we identified Unc-51-like kinase 1 (ULK1) as a novel positive regulator of type I IFN signaling, which is essential for both type I IFN-induced anti-viral and anti-proliferative responses [4]. Additionally, we presented evidence showing that engagement of type I IFN receptor activates a novel pathway, possibly involving both PI3K-AKT-dependent and independent meditators, culminating in activation of ULK1 [4]. Importantly, ULK1 kinase activity seems to cooperate, either directly or through intermediate kinases, with the MAPKKK-MKK3/6 signaling cascade for optimal IFN-induced activation of p38 MAPK [4]. Thus, ULK1 activation appears to mediate transcription of ISGs, in part, via regulation of the p38 MAPK pathway [4]. In a recent report, ULK1 was shown to localize to the nucleus and promote cell death by increasing poly (ADP-ribose) polymerase 1 (PARP1) activity in response to reactive oxygen species-induced cellular damage [5]. These new findings, taken together with our recently published report [4], raise the intriguing possibility that ULK1 translocates into the nucleus in response to type I IFNs, and could directly or indirectly control transcription of ISGs, but this remains to be explored in future studies.

Type I IFNs can be produced by all nucleated cell types in response to pathogen infection, physiological signals, and other inducers and stimuli [6, 7]. However, the induction of an IFN response involves expression and activation of both positive and negative regulators of Type I IFN production and signaling [6, 7]. Many of these negative regulators are ISGs and IFN-regulated proteins, which control the time and magnitude of an IFN response, preventing inflammation and tissue damage [6, 7]. Interestingly, this seems to be the case also for ULK1 [8]. The stimulator of IFN genes (STING) is activated by cyclic dinucleotides, leading to activation of TBK1 (TRAF family member associated NF-κB activator-binding kinase 1) and phosphorylation of IRF3 [6, 7]. Active IRF3 translocates into the nucleus and induces expression of IFN genes, initiating an IFN response [6, 7]. Our data suggests that ULK1 is activated downstream of the type I IFN receptor, leading to transcription of key ISGs and positively mediating IFN responses [4]. However, based on other recent work, at a later stage ULK1 is activated by cyclic dinucleotides and acts as a negative regulator of STING, resulting in inhibition of IRF3, and subsequent blockage of type I IFN production, thus shutting down the IFN response [8]. Thus, taking together the evidence presented by Kono *et al*. [8] and our findings [4], one can propose a model in which ULK1 may initially promote IFN responses against pathogens, but it later inhibits STING activity and IFN production, thus limiting/optimizing the IFN response (Figure 1). Further studies are warranted to fully understand this dual role of ULK1 in the IFN system. This has important implications in the IFN-signaling field and may provoke other questions in the future. For example, it would be interesting to identify putative phosphorylation sites of ULK1 that may correlate with negative versus positive regulatory effects on Type I IFN responses. A complete understanding of the mechanisms of action of ULK1 during IFN-signaling may also prove fundamental for the development of better therapies designed to either promote or inhibit type I IFN-dependent responses.

**Figure 1:**
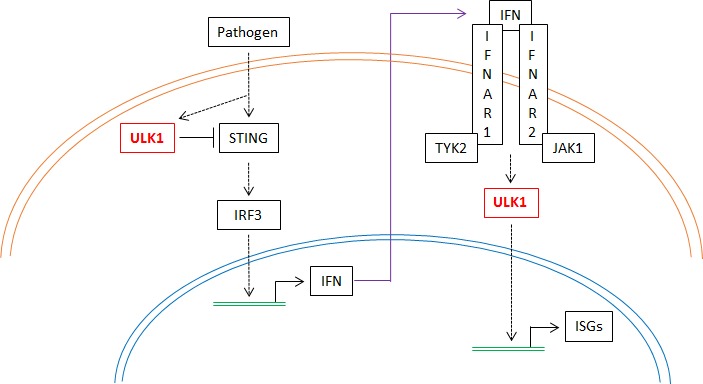
Regulatory roles of ULK1 in Type I IFN responses

## References

[1] Platanias LC (2005). Nat Rev Immunol.

[2] Fish EN, Platanias LC (2014). Mol Cancer Res.

[3] Saleiro D, Platanias LC (2015). Trends Immunol.

[4] Saleiro D (2015). Cell Rep.

[5] Joshi A (2015). Cell Death Differ.

[6] Schneider WM (2014). Annu Rev Immunol.

[7] Porritt RA, Hertzog PJ (2015). Trends Immunol.

[8] Konno H (2013). Cell.

